# Intersection of alcohol use, pain symptoms, and negative affect in total knee arthroplasty patients and people with HIV

**DOI:** 10.1016/j.jpain.2025.105446

**Published:** 2025-05-20

**Authors:** Taylor Fitzpatrick-Schmidt, Vinod Dasa, Claudia Leonardi, Tekeda F. Ferguson, David A. Welsh, Patricia E. Molina, Martin J.J. Ronis, Scott Edwards

**Affiliations:** a Comprehensive Alcohol-HIV/AIDS Research Center, Louisiana State University Health Sciences Center, New Orleans, LA 70112, USA; b Department of Orthopedics, School of Medicine, Louisiana State University Health Sciences Center, New Orleans, LA 70112, USA; c School of Public Health, Louisiana State University Health Sciences Center, New Orleans, LA 70112, USA

**Keywords:** Alcohol, Pain, Total knee arthroplasty, HIV, Negative affect

## Abstract

Alcohol is a well-known analgesic, although excessive alcohol use can lead to hyperalgesia and heightened negative affect. This cross-sectional study examined associations between alcohol use, self-reported pain, and negative affective symptoms in two distinct cohorts of patients vulnerable to chronic pain: individuals undergoing total knee arthroplasty (TKA) surgery and a cohort of people with HIV (PWH). Participants were enrolled in two clinical studies: a retrospective study of patients undergoing TKA and a longitudinal study of PWH, the New Orleans Alcohol Use in HIV (NOAH) cohort. Based on the Alcohol Use Disorders Identification Test – Consumption (AUDIT-C) score, participants in both cohorts were stratified as alcohol drinkers (AUDIT-C ≥ 1) or non-drinkers (AUDIT-C < 1). In the NOAH cohort, pain intensity and interference were assessed using the 36-Item Short Form Survey (SF-36). In the TKA cohort, self-reported pain was quantified using the Pain Intensity and Pain Interference items from the Patient-Reported Outcomes Measurement Information System^®^ (PROMIS-29) and the Knee Osteoarthritis and Outcomes Score (KOOS) Pain scores. Alcohol drinkers reported fewer pain symptoms compared to non-drinkers across both cohorts, and females with HIV reported more pain compared to males with HIV. Furthermore, pain symptoms were associated with increased anxiety and depression symptoms in both cohorts, and in PWH these associations appeared stronger in drinkers compared to non-drinkers. These findings suggest that although alcohol may offer some analgesic benefits, patients should be cautioned about its use for self-medication, as it may increase risk for pain-related negative affective comorbidities.

**Perspective:**

Alcohol can both relieve and exacerbate pain. The current study discovered that ongoing alcohol use was associated with fewer self-reported pain symptoms but appeared to increase associations between pain and negative affective symptoms in two vulnerable cohorts. Findings support cautioning patients against the recurring use of alcohol for pain management.

## Introduction

Alcohol has been used as an analgesic for centuries and is commonly self-administered for pain relief.^1–3^ Despite its immediate analgesic efficacy, chronic alcohol use can contribute to the development of hyperalgesia, particularly during abstinence^4–6^ (Edwards et al., 2020; Dina et al., 2008; Edwards et al., 2012). Furthermore, patients suffering from alcohol use disorder (AUD) often develop a painful neuropathy, a serious comorbidity caused by alcohol-induced nerve damage.^4,7,8^ This suggests a bidirectional relationship in which alcohol influences pain, and chronic pain, in turn, drives alcohol consumption.^4^

Negative affective symptoms (e.g., anxiety, depression) are key factors in this relationship between pain and alcohol use, as they contribute to increased alcohol consumption and relapse risk.^9–11^ Negative affective disorders such as general anxiety disorder and major depressive disorder are highly comorbid with chronic pain, and patients with both conditions report increased pain severity and pain interference.^12,13^ These relationships may be particularly detrimental for people with HIV (PWH), a population with a high prevalence of chronic pain and negative affective comorbidities.^14^ Similarly, patients undergoing surgery face an elevated risk of chronic post-surgical pain, making them another group vulnerable to pain-related alcohol use.^15^

Sex assigned at birth is an important factor in both AUD and chronic pain risk.^16^ Indeed, people assigned female at birth (AFAB) are more sensitive to pain and more likely to develop chronic pain conditions.^17,18^ Although AUD has historically been more common among people assigned male at birth (AMAB), rates of alcohol use and AUD in recent years are rising among AFABs.^19^ Furthermore, AFABs are more susceptible to sequelae of AUD and may progress to AUD faster than AMABs, a phenomenon known as the “telescoping effect”.^19–21^ Despite these disparities, most research has historically focused on AMABs.^17^ Given these sex differences, comparing the relationship between pain and alcohol use in both AMABs and AFABs is important to determine if there are sex differences at the intersection of pain, alcohol, and negative affect. Due to the unavailability of gender identity data, this study uses the terms “female” and “male” to refer to AFABs and AMABs, respectively.

This cross-sectional study analyzed two distinct at-risk populations: individuals undergoing total knee arthroplasty (TKA) and PWH enrolled in the New Orleans Alcohol Use in HIV (NOAH) Study. Both cohorts consist of patients vulnerable to chronic pain, which may drive increased alcohol consumption and increase AUD risk.^14,22^ To strengthen the validity of these findings, two distinct cohorts were included, which allows the assessment of the replicability of these results across two different populations with varying demographic characteristics. The primary aim was to examine associations between pain, negative affect, and alcohol use in these two large, diverse cohorts. The hypothesis was that 1) alcohol use, as assessed by AUDIT-C, would be associated with reduced pain symptoms and 2) pain would be associated with increased anxiety and depression symptoms in both cohorts. Additionally, it was predicted that drinking status and sex would influence these associations, hypothesizing that drinkers would show stronger associations between pain and negative affect and that females would report more pain, anxiety, and depression symptoms than males.

## Methods

### Total Knee Arthroplasty (TKA) cohort characteristics and survey instruments

This cross-sectional analysis used data from a retrospective cohort study conducted on consecutive patients who underwent primary unilateral total knee arthroplasty (TKA) by a single fellowship-trained orthopedic surgeon between October 2017 and June 2021, using an enhanced recovery TKA protocol. The study was reviewed and approved by the Louisiana State University Health Sciences Center – New Orleans (LSUHSC-NO) Institutional Review Board and all subjects provided informed consent. Enrolled participants (n=380) completed a pre-surgery visit during which data was collected via survey questionnaires and physical exam. Study sample size was determined by power analysis and data availability. Self-administered surveys were collected through LSUHSC-NO’s online REDCap^™^ (Research Electronic Data Capture) System. Pain was assessed using the Pain Intensity item from the Patient-Reported Outcomes Measurement Information System^®^ (PROMIS-29^®^), which uses a 0–10 numerical pain rating scale with a lower score indicating less pain, the PROMIS Pain Interference scale,^23^ which uses a scale of 20–80 with a lower score indicating less pain, and the Knee Osteoarthritis and Outcomes Score (KOOS) Pain subscale score, which uses a scale of 0–45 where a higher score indicates less pain.^24^ Furthermore, the PROMIS Anxiety and Depression scales were used to assess anxiety and depressive symptoms in the TKA cohort.^23^ Alcohol use was assessed using the consumption component of the Alcohol Use Disorders Identification Test (AUDIT-C) questionnaire, scored on a scale from 0–12 with a higher score indicating increased alcohol consumption and higher risk of developing an AUD. In both cohorts, alcohol drinkers were defined as AUDIT-C ≥ 1.

### New Orleans alcohol use in HIV (NOAH) cohort characteristics and survey instruments

Data collected at baseline from a cohort of 365 people with HIV (PWH) enrolled in the New Orleans Alcohol Use in HIV (NOAH) study, was used for this cross-sectional analysis. Participants were recruited from local HIV clinics from October 2015 to November 2017. Findings have been disseminated to the broad public, including scientific and lay audiences, for feedback and guidance on future directions. Additionally, the Outreach and Information Dissemination Core of the NIH-funded Comprehensive Alcohol HIV AIDS/Research Center (CARC) will continue to engage with the public as part of this longitudinal study, including through interactions with the Community Advisory Board. Additional details on study parameters and participant demographics have been described previously.^25^ The study was approved by the LSUHSC-NO Institutional Review Board and all subjects provided informed consent. Enrolled participants completed a baseline visit during which data were collected through survey questionnaires, physical exam, function performance tests, and biological specimen and blood collection. Interviewer-administered surveys were collected through LSUHSC-NO’s online REDCap^™^ System by trained research staff. Pain was assessed using the self-reported 36-Item Short Form Survey (SF-36) pain scale (RAND Corporation, Ware and Sherbourne, 1992), which includes separate measures for pain intensity and pain interference (i.e., how pain interferes with activities of daily living). For both pain measures, the scale is 0–100, with lower scores indicating more pain or pain interference. Anxiety and depressive symptoms were measured using the Hospital Anxiety and Depression Scale (HADS), a validated and reliable screening tool consisting of 14 items, including seven item subscales for both anxiety and depression, scored from 0–21 with higher scores indicating more anxiety and depressive symptoms.^26,27^ Alcohol use in the NOAH cohort was also assessed using AUDIT-C, and alcohol drinkers were defined as AUDIT-C ≥ 1.

### Statistical analyses

Data from the TKA Cohort was analyzed using SAS version 9.4 (SAS Institute Inc, Cary, NC, USA), while data from the NOAH study was analyzed using SAS OnDemand for Academics. Based on the distribution of AUDIT-C scores in both cohorts (see Supplementary Figure 1A–E), a cut-off score of 1 was applied to classify participants as drinkers (AUDIT-C ≥ 1) and non-drinkers (AUDIT-C = 0). Descriptive statistics were calculated for participant demographics and outcome (pain and negative affect) variables for both cohorts by drinking status. Continuous variables were compared using two-sample Student’s t-tests, while categorical variables were assessed using Pearson’s chi-square tests.

To address the first hypothesis, associations between drinking status and outcome variables (pain and negative affect) were examined using two-sample Student’s t-tests. Additionally, mixed linear models (PROC MIXED) were employed to further evaluate these relationships, with models adjusted for sex and race which were significantly different between the two groups to reduce potential confounders. Mixed linear models for the NOAH cohort were also stratified by smoking status. Spearman correlation coefficients were calculated to examine associations between pain and negative affect (anxiety and depression symptoms) and to determine whether these associations differed by sex and drinking status using Fisher’s z-transformation. Finally, Spearman correlations were conducted to analyze the relationship between AUDIT-c as a continuous variable and all pain variables across both cohorts.

Figures were generated using Prism 10 (GraphPad Software, Inc., La Jolla, CA, USA). When applicable, model residuals were assessed to ensure they were independent, identically distributed, and normally distributed with homogenous variances. Statistical significance was determined using a two-sided alpha level of less than 0.05.

## Results

### Patient characteristics of the NOAH and TKA cohorts

Data from 365 subjects enrolled in the NOAH study were included in this cross-sectional analysis, with 314 participants categorized as alcohol drinkers (AUDIT-C ≥ 1) and 51 participants categorized as non-drinkers (AUDIT-C=0) (Table 1). Complete baseline demographic information for all NOAH participants has been published previously (Welsh et al., 2019). In this study, drinkers and non-drinkers differed by sex (p=0.043) and race (p=0.042). In the TKA cohort, a total of 380 participants were included in the study, out of which 234 were categorized as alcohol drinkers (AUDIT-C ≥ 1) and 146 as non-drinkers (AUDIT-C=0) (Table 2). Similar to the NOAH cohort, drinkers and non-drinkers in the TKA cohort also differed by sex (p<0.0001) and race (p=0.036).

### Alcohol use is associated with reduced pain symptoms in people with HIV

**Fig. 1.**
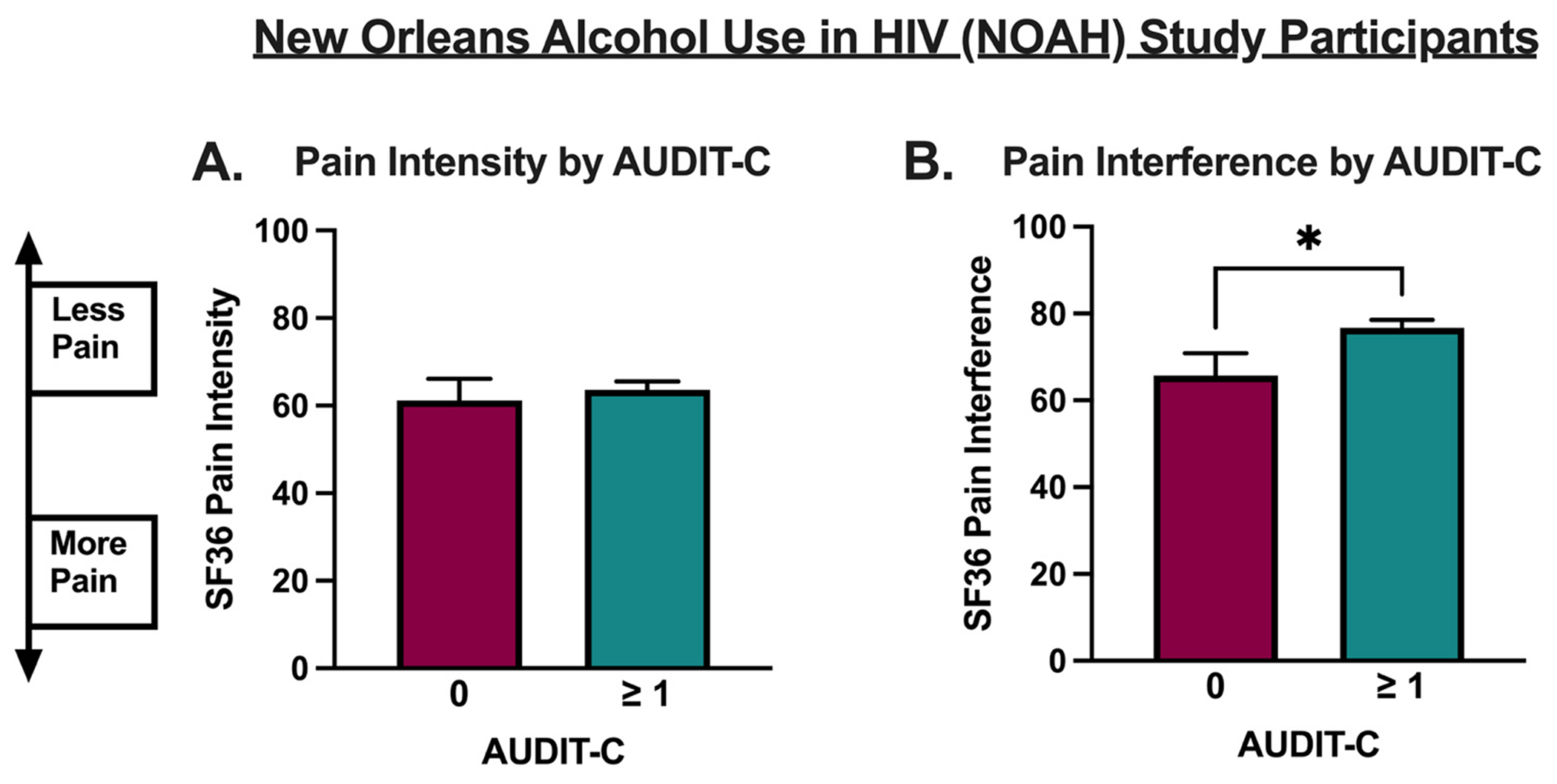
36-Item Short Form Survey (SF-36) pain scores stratified by alcohol use among New Orleans Alcohol Use in HIV (NOAH) participants. A. SF-36 pain intensity scores did not differ between drinkers (AUDIT-C ≥ 1) and non-drinkers (AUDIT-C < 1). B. SF-36 pain interference scores were significantly higher (less pain) in drinkers compared to non-drinkers when stratified by AUDIT-C scores. Data were analyzed using unpaired t-tests, * P < 0.05.

NOAH participant outcomes are listed in Table 1. In the NOAH cohort, SF-36 pain intensity scores were higher (less pain) for alcohol drinkers (AUDIT-C ≥ 1) compared to non-drinkers (AUDIT-C = 0), although this difference did not reach statistical significance (p=0.648, t=0.4567, df=362, Fig. 1A); however SF-36 pain interference scores were significantly higher (less pain interference) among alcohol drinkers with AUDIT-C ≥ 1 compared to non-drinkers (p=0.027, t=2.228, degrees of freedom (df)=362, Fig. 1B). Multivariable models revealed SF-36 pain interference scores remained significantly higher (less pain interference) in alcohol drinkers (AUDIT-C ≥ 1) after adjusting for sex, race, and smoking status (p=0.032, F(_1357_) = 4.62, Table 3). Supplementary Table 1 presents exploratory analyses of the relationship between continuous AUDIT-C scores and pain variables across both cohorts, using unadjusted and adjusted Spearman correlations.

### Alcohol use is associated with reduced pain symptoms in TKA patients

**Fig. 2.**
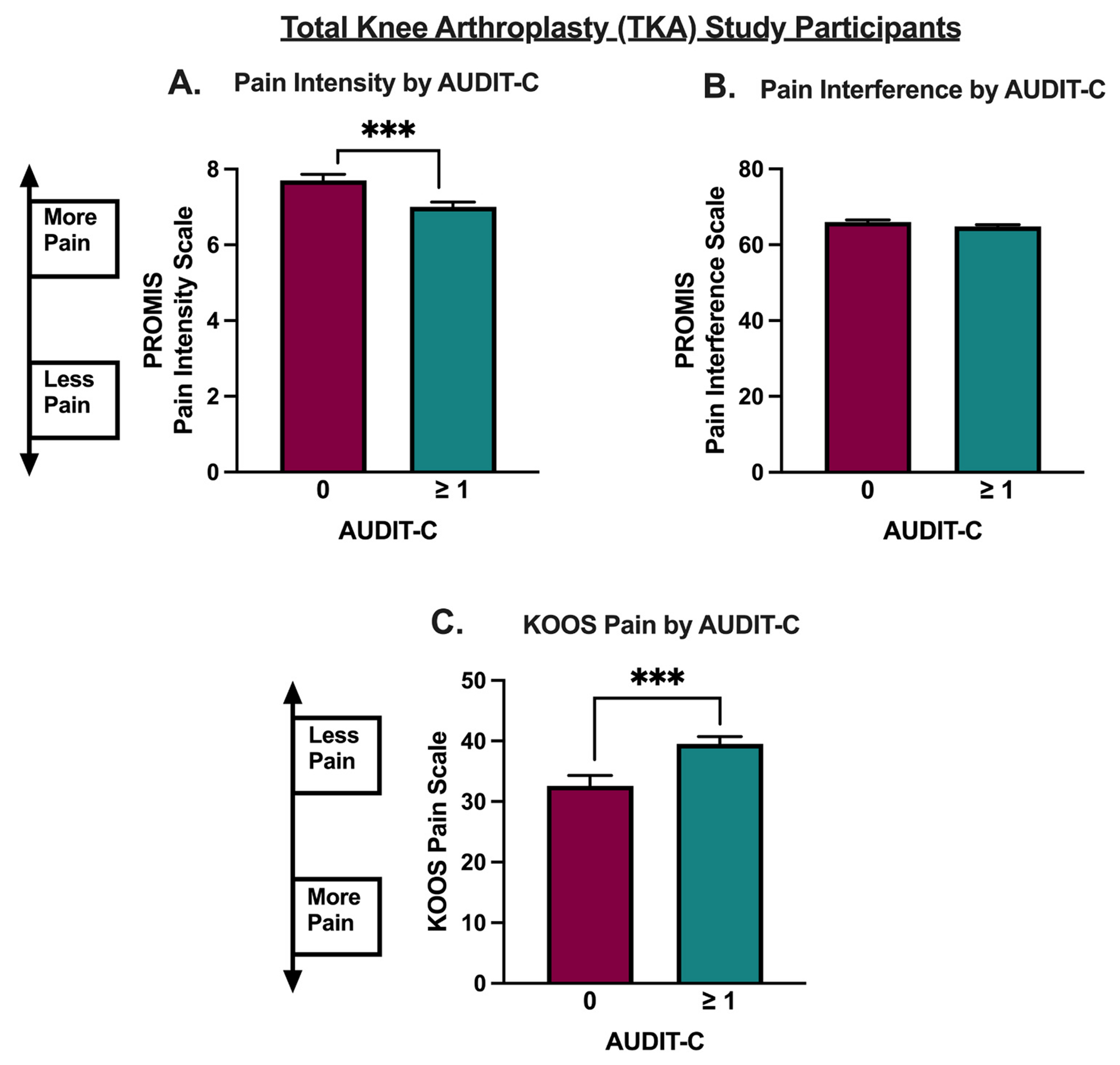
Patient-Reported Outcomes Measurement Information System (PROMIS-29) and Knee Osteoarthritis and Outcomes Score (KOOS) pain scores stratified by alcohol use among Total Knee Arthroplasty (TKA) patients. A. PROMIS-29 pain intensity scores were significantly lower (less pain) in alcohol drinkers (AUDIT-C ≥ 1) compared to non-drinkers (AUDIT-C < 1). B. PROMIS-29 pain interference scores were not significantly different between alcohol drinkers and non-drinkers. C. KOOS pain scores were significantly higher (less pain) in drinkers compared to non-drinkers. Data were analyzed using unpaired t-tests, *** P < 0.001.

TKA participant outcomes are listed in Table 2. In the TKA cohort, alcohol drinkers with AUDIT-C ≥ 1 reported significantly less pain compared to non-drinkers using the PROMIS-29 (where a lower score is indicative of less pain) pain score (p=0.003, t=3.319, df=378, Fig. 2A) and although alcohol drinkers had numerically lower scores using the PROMIS-29 Pain Interference scale (where a lower score is indicative of less pain), this decrease did not reach statistical significance (p=0.111, t=1.556, df=378, Fig. 2B). Using the KOOS pain score, alcohol drinkers also had higher scores (less pain) compared to non-drinkers (p=0.0009, t=3.370, df=378, Fig. 2C). Multivariable models revealed PROMIS-29 pain scores remained significantly lower (less pain; p=0.017, F(_1367_) = 5.81) and KOOS pain scores remained significantly higher (less pain; p=0.010, F_(1364)_ = 6.66) in alcohol drinkers after adjusting for sex and race (Table 3).

### Negative affective symptoms are associated with pain in both cohorts

We next analyzed associations between pain and anxiety and depressive symptoms in the NOAH and TKA cohorts. In the NOAH cohort, both SF-36 pain intensity and pain interference scores were significantly associated with anxiety and depression scores, as assessed by the Hospital Anxiety and Depression Scale (HADS; Table 4; p<0.0001). In the TKA cohort, all three pain scores were significantly associated with anxiety and depression scores, as assessed by the PROMIS-29 (Table 5; p<0.0001). Data indicate that more pain symptoms are associated with more anxiety and depressive symptoms across both cohorts, although the direction of the association differs based on nature of the scale.

### Females with HIV in the NOAH cohort report more pain symptoms

**Fig. 3.**
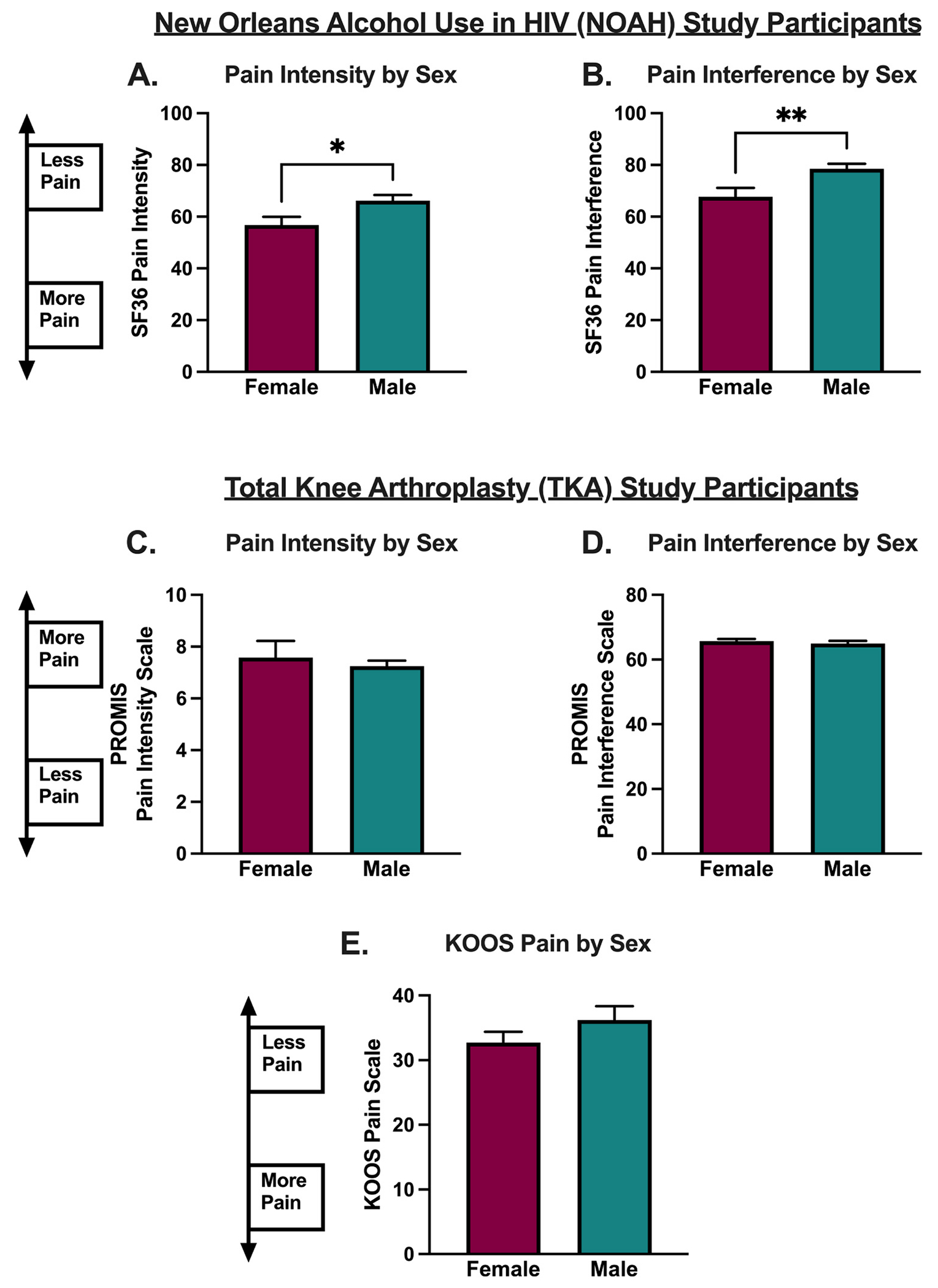
Pain scores stratified by sex among New Orleans Alcohol Use in HIV (NOAH) participants and Total Knee Arthroplasty (TKA) participants. A. 36-Item Short Form Survey (SF-36) pain intensity scores were significantly lower (more pain) in females compared to males in the NOAH cohort. B. SF-36 pain interference scores were significantly lower (more pain) in females compared to males in the NOAH cohort. C. Patient-Reported Outcomes Measurement Information System (PROMIS-29) pain intensity scores were not significantly different when stratified by sex in the TKA cohort. D. PROMIS-29 pain interference scores were not significantly different when stratified by sex in the TKA cohort. E. Knee Osteoarthritis and Outcomes Score (KOOS) pain scores were not significantly different when stratified by sex in the TKA cohort. Data were analyzed using unpaired t-tests, * P < 0.05, ** P <0.01.

**Fig. 4.**
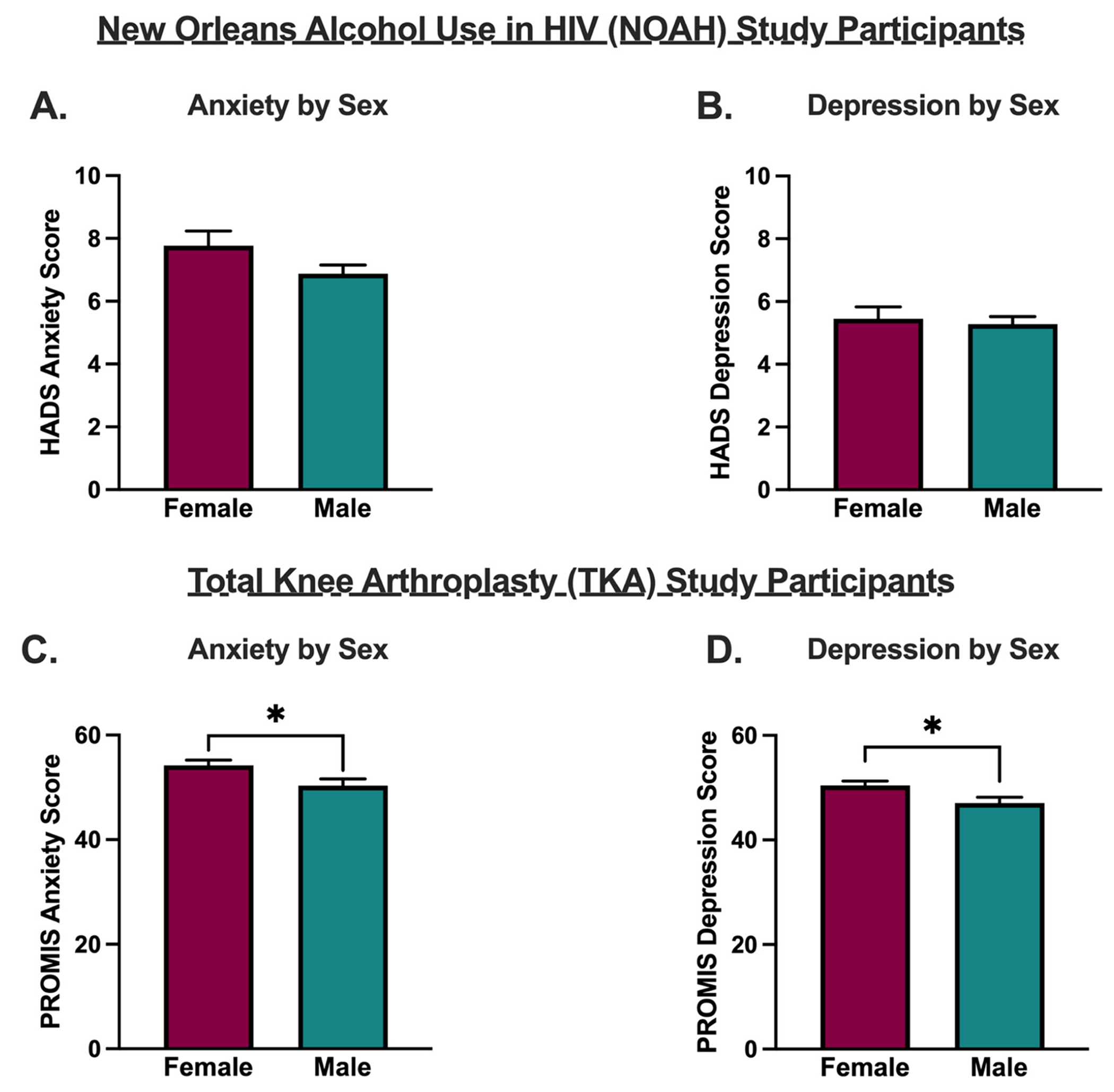
Anxiety and depression scores stratified by sex among New Orleans Alcohol Use in HIV (NOAH) participants and Total Knee Arthroplasty (TKA) participants. A. Hospital Anxiety and Depression (HADS)-Anxiety scores were not significantly different among males and females in the NOAH cohort. B. HADS-Depression scores were not significantly different among males and females in the NOAH cohort. C. Patient-Reported Outcomes Measurement Information System (PROMIS-29) anxiety scores were not significantly higher in females in the TKA cohort. D. PROMIS-29 depression scores were significantly higher in females in the TKA cohort.

Finally, sex differences in pain and negative affective symptoms were examined in both cohorts. In the NOAH cohort, females reported significantly more pain (p=0.017, t=2.398, df=362, Fig. 3A) and pain interference (p=0.004, t=2.933, df=362, Fig. 3B) compared to males, while in the TKA cohort, pain scores did not differ by sex (PROMIS Pain Intensity: p=0.737, t=0.3367, df=370, Figure 3C; PROMIS Pain Interference: p=0.442, t=0.7701, df=362, Fig. 3D; KOOS Pain: p=0.216, t=1.240, df=367, Fig. 3E), Furthermore, in the NOAH cohort, anxiety and depression scores, as assessed by the Hospital Anxiety and Depression Scale (HADS), did not differ by sex (HADS-Anxiety: p=0.083, t=1.740, df=362; Fig. 4A; HADS-Depression: p=0.701, t=0.3846, df=363; Fig. 4B), while in the TKA cohort, PROMIS anxiety and depression scores were significantly higher in females (more anxiety and depression symptoms) compared to males (PROMIS-Anxiety: p=0.024, t=2.272, df=364, Fig. 4C; PROMIS-Depression: 0.022, t=2.302, df=363; Fig. 4D).

### Individual associations among pain, anxiety, and depression symptoms in drinking vs. non-drinking female and Male PWH and TKA patients

Given the divergent sex-dependent relationships between recent drinking and negative affective symptoms in PWH, correlative relationships across pain, negative affect, and drinking status in male and female PWH (Table 6) and TKA patients (Table 7) were assessed. In Table 6, negative correlations represent positive associations due to the inverted nature of the SF-36 scale. In the NOAH cohort, female drinkers with AUDIT-C ≥ 1 displayed a significant positive association between pain intensity and anxiety symptoms, while female non-drinkers had significant positive associations between pain interference and anxiety symptoms (Table 6). In contrast, male drinkers with AUDIT-C ≥ 1 displayed positive associations between pain intensity and pain interference and both anxiety and depression symptoms, and this was not observed in male non-drinkers (Table 6).

In Table 7, positive correlations represent positive associations for PROMIS-29 pain scores, while negative correlations represent positive associations for KOOS pain scores due to the inverted nature of the KOOS scale. In the TKA cohort, females displayed positive associations between all three pain measures and both anxiety and depression symptoms, regardless of drinking status, while associations between pain and anxiety and depression symptoms were only seen among male drinkers (Table 7).

## Discussion

These data demonstrate that alcohol drinkers report fewer pain symptoms compared to non-drinkers across two cohorts of vulnerable patients at high risk for chronic pain, including a cohort of people with HIV and a cohort of patients undergoing total knee arthroplasty surgery (Figs. 1 and 2). These data are consistent with several studies that have shown light and moderate alcohol consumption are associated with reduced pain symptoms.^3,28^ For example, one previous report found that moderate drinkers have improved Quality of Well-being Index (QWB-7) scores, SF-36 pain scores, Western Ontario and McMaster Universities Arthritis Index (WOMAC) scores, and a shorter length of stay following knee arthroplasty compared to non-drinkers.^29^ A second study found that moderate alcohol consumption is associated with significantly reduced pain and fibromyalgia symptoms in patients with chronic pain.^30^ Another study in a cohort of fibromyalgia patients, found that lower and moderate alcohol consumption was associated with fewer fibromyalgia symptoms and improved quality of life compared to non-drinkers and heavy drinkers.^31^ Thus, the findings from the present study are consistent with the existing literature and extend these findings to a cohort of PWH.

In contrast to the cohort demographics of the knee surgery and fibromyalgia cohorts in previous studies, consisting of older patients (50–70) who are predominantly female and white, participants enrolled in the NOAH cohort are younger (20–60), predominantly (69%) male, and predominantly (84%) Black. Despite these major demographic differences, the association between alcohol consumption and reduced pain is consistent across both cohorts, suggesting the analgesic effects of alcohol consumption are largely independent of age, sex, and race. These data further support the concept that light-to-moderate alcohol consumption might offer certain benefits relative to abstention, although future studies should focus on differentiating light, moderate, and heavy alcohol consumption to better understand this shift.^32^ Importantly, direct laboratory assessments have also described the effects of alcohol to reduce pain and pain-related negative affect.^33^

In line with previous work, these results suggest important sex differences in the relationship between pain, negative affect, and alcohol consumption in PWH. A significant main effect of sex was found only in the NOAH cohort of PWH, where females reported overall increased pain intensity and pain interference compared to males. This is consistent with the majority of existing literature on sex differences in pain, which indicate increased pain sensitivity and increased risk of pain chronification in females.^34–36^ Sex-dependent relationships among drinking status, pain, and negative affect in PWH were also discovered (Table 6). These findings resonate with previous work demonstrating that psychosocial factors may exacerbate chronic pain symptoms in PWH,^30^ and that PWH with more severe pain report more mood-related impairments.^37^ Interestingly, in females with HIV, pain intensity was significantly and positively associated with anxiety symptoms only in drinkers, while pain interference was significantly and positively associated with anxiety symptoms in non-drinkers. In comparison, male drinkers with HIV (but not non-drinkers) displayed significant positive associations between pain and depression symptoms. These findings suggest that alcohol use may link pain symptoms and specific negative affective conditions in a sex-dependent manner in PWH, further highlighting the need for the study and therapeutic targeting of psychiatric transdiagnostic factors in these areas as they relate to AUD risk.^38–40^ In the TKA cohort, females displayed positive associations between pain and both anxiety and depression symptoms, regardless of drinking status, while associations between pain and anxiety and depression were seen among male drinkers but not male non-drinkers.

Although prominent sex differences were not observed in pain reported by the TKA participants, this may be due to the low number of female alcohol drinkers (n=9) in this study population. Importantly, data analysis for both groups was limited to the inclusion of biological sex, as gender identity was not available as a variable for either study. Future studies should include gender identity to better understand the distinct contributions of sex and gender to the intricate relationship between pain and alcohol consumption. Another limitation present in the current study include the cross-sectional nature of the study, which precludes the ability to draw conclusions regarding the causality of the decreased pain in alcohol drinkers, which could be attributed to fewer comorbidities in drinkers.^41^ Furthermore, the cross-sectional nature of the study limits the ability to investigate the bidirectional nature of the associations between negative affective symptoms, pain, and alcohol use. Future longitudinal studies will be necessary to assess the temporal relationships between these variables and determine whether alcohol use mediates the associations between pain and negative affective symptoms. As research progresses and sufficient power is achieved for these analyses, further investigate of these complex relationships is planned.

Additionally, this study used cut-offs to categorize participants as “drinkers” or “non-drinkers”, which limits the ability to distinguish the impact of light, moderate, and heavy alcohol consumption on pain symptoms. Unfortunately, these categorizations were not possible in the present study due to small sample sizes in both cohorts and the distribution of the AUDIT-C scores in both cohorts. Future work should focus on better delineating these effects, as predict chronic heavy alcohol consumption is predicted to exacerbate pain symptoms and contribute to the development of hyperalgesia.^42,43^ A key limitation is the self-report nature of the pain measures and AUDIT-C, and in both cohorts, patients were not classified as life-long abstainers or previous drinkers, which may also impact interpretation of these results. Finally, various medications participants may be taking could influence pain, anxiety, and depression. Unfortunately, accurate data on medication use were not available for these studies, limiting the ability to control for these variables in the present analyses. Future studies with access to such data should aim to address this limitation.

From a mechanistic perspective, there are several possible explanations underlying fewer pain symptoms in alcohol drinkers. One such mechanism is through endogenous opioid signaling, as there is evidence that opioid receptor signaling may contribute to both the reinforcing and analgesic effects of alcohol.^44,45^ The endocannabinoid system has also been shown to be implicated in the analgesic effects of alcohol, specifically mediated by cannabinoid receptor type 1 (CB1R) signaling.^13^ In addition, it is possible that ethanol’s action as a central nervous system depressant and GABA receptor agonist may underlie its analgesic effects.^46^ In the context of AUD and several chronic pain conditions, the balance of excitatory and inhibitory neurotransmitters is altered with increased excitatory glutamate and reduced inhibitory GABA levels.^42^ These effects are negated by acute ethanol treatment, which enhances GABAergic neurotransmission and reduces glutamate channel activity.^47^ In addition, there is evidence that moderate alcohol consumption is associated with reduced inflammation in diabetic men^48^ and in mouse models of arthritis^49^ that might contribute to protection against tissue injury, potentially contributing to reduced pain in alcohol drinkers. Moreover, in the case of TKA, there are data from a meta-analysis of epidemiological studies that alcohol consumption (up to 2 drinks per day) is associated with increased bone mineral density relative to non-drinkers, whereas higher levels of consumption increase fracture risk.^50^ Finally, other behavioral and sociocultural factors may contribute to the analgesic effects of alcohol. For example, a family history of AUD can influence analgesia in the context of alcohol use.^51^ Thus, factors beyond the pharmacological mechanisms of alcohol’s analgesic effects should be considered.

In conclusion, the present study supports the analgesic efficacy of alcohol use and evidence of reduced pain symptoms in alcohol drinkers in two distinct cohorts, one of patients undergoing total knee arthroplasty (TKA) and the other of people with HIV (PWH). Furthermore, females with HIV reported increased pain intensity and interference. Regardless of sex, alcohol consumption, as assessed by AUDIT-C, was associated with reductions in pain, suggesting that the relationship between alcohol consumption and pain mitigation are independent of pain source, age, sex, and ethnicity. Nonetheless, despite possible analgesic benefits, health providers should caution patients about the use of alcohol for pain relief given the dangers of chronic alcohol exposure over time on multiple organ systems and psychiatric disease processes.^52,53^ Given the growing prevalence of chronic pain conditions, there is an urgent need to develop effective pain medications that represent safer alternatives to abused substances like alcohol. These findings further highlight the need for integrative mental health screenings for alcohol use, pain symptoms, and negative affect in vulnerable populations, including chronic pain patients and PWH.^54^

## Supplementary Material

Supplementary Figure 1

Supplementary Table 1

## Figures and Tables

**Table 1:** Patient baseline demographics and clinical characteristics for New Orleans Alcohol Use in HIV (NOAH) Cohort.

Characteristic	Non-Drinkers (AUDIT-C=0) (n=51)	Alcohol Drinkers (AUDIT-C≥1)(n=314)	P value
Age (years), mean (SD)	49.3 (9.8)	48.1 (10.4)	0.416
BMI (kg/m^2^), mean (SD)	28.4 (6.0)	27.0 (7.1)	0.193
Sex, % (n)			0.043
Male	56.9 (29)	71.0 (223)	
Female	43.1 (22)	29.0 (91)	
Race, % (n)			0.042
Black or African American	72.6 (37)	85.4 (268)	
White or Caucasian	25.5 (13)	14.0 (44)	
Other	2.0 (1)	0.6 (2)	
Viral load category, % (n)			1.000
<400 copies/mL	87.5 (42)	87.1 (269)	
400–5000 copies/mL	4.2 (2)	4.6 (14)	
>5000 copies/mL	8.3 (4)	8.4 (26)	
Smoking category			0.095
Never Former Current	25.5 (13)25.5 (13)49.0 (25)	22.6 (71)62.7 (197)14.7 (46)	
CD4 count	591.5 (313.9)	428.0 (305.0)	0.183
SF–36, mean (SD)			
Pain intensity	61.2 (35.4)	63.6 (34.7)	0.648
Pain interference HADS, mean (SD)	65.7 (36.7)	76.7 (32.0)	0.027
Anxiety	6.9 (4.6)	5.9 (4.5)	0.628
Depression	5.9 (4.5)	5.2 (3.8)	0.230

BMI = body mass index; SD = standard deviation; SF-36 = 36-Item Short Form Survey; HADS = Hospital Anxiety and Depression Scale

**Table 2:** Patient baseline demographics and clinical characteristics for Total Knee Arthroplasty (TKA) Cohort.

Characteristic	Non-Drinkers (AUDIT-C=0) (n=146)	Alcohol Drinkers (AUDIT-C≥1)(n=234)	P value
Age (years), mean (SD)	68.2 (9.3)	66.9 (8.9)	0.178
BMI (kg/m^2^), mean (SD)	33.0 (6.3)	32.9 (6.1)	0.917
Sex, % (n)			<0.0001
Male	19.9 (29)	39.3 (92)	
Female	80.1 (117)	60.7 (142)	
Race, % (n)			0.036
Black or African American	37.7 (55)	33.8 (79)	
White or Caucasian	53.4 (78)	62.8 (147)	
Other	8.9 (13)	3.4 (8)	
Insurance type, % (n)			0.691
Private	34.2 (50)	36.3 (85)	
Medicare	17.8 (26)	21.0 (49)	
Medicaid	19.2 (28)	14.1 (33)	
Medicare	27.4 (40)	26.5 (62)	
Advantage			
Other	1.4 (2)	2.1 (5)	
Smoking category, % (n)			0.220
Every day	8.3 (12)	7.3 (17)	
Some days	1.4 (2)	4.7 (11)	
Not at all	90.3 (130)	88.0 (206)	
PROMIS–29, mean (SD)			
Anxiety	52.3 (12.1)	51.3 (11.2)	0.468
Depression	48.3 (9.6)	48.7 (10.0)	0.688
Pain Interference	66.0 (7.0)	64.8 (7.5)	0.111
Pain	7.7 (2.0)	7.0 (2.0)	0.003
KOOS - Pain, mean (SD)	32.6 (20.8)	39.5 (18.5)	0.0009

BMI = body mass index; KOOS = Knee injury and Osteoarthritis Outcome Score; PROMIS = Patient-Reported Outcomes Measurement Information System; SD = standard deviation

**Table 3:** Multivariable adjusted participant-reported outcomes, including pain intensity, pain interference, anxiety and depression, for non-drinkers and drinkers in the New Orleans Alcohol Use in HIV (NOAH) and Total Knee Arthroplasty (TKA) cohorts.

Item	Non-Drinkers (AUDIT-C=0)	Alcohol Drinkers (AUDIT-C≥1)	F-value _(df1 df2)_	p-value
NOAH cohort	(n=51)	(n=314)		
SF–36				
Pain Intensity	68.3 (8.0)	69.0 (7.0)	F_(1357)_ = 0.01	0.917
Pain Interference	69.4 (7.5)	79.1 (6.6)	F_(1357)_ = 4.62	0.032
HADS				
Anxiety Depression	7.5 (1.1)5.6 (0.9)	8.1 (0.9)4.9 (0.8)	F_(1358)_ = 0.39 F_(1358)_ = 2.01	0.5300.157
TKA cohort	(n=146)	(n=234)		
PROMIS–29				
Anxiety	52.1 (1.2)	52.4 (1.1)	F_(1361)_ = 0.04	0.836
Depression	48.0 (1.0)	49.4 (0.9)	F_(1360)_ = 1.70	0.194
Pain Interference	65.8 (0.8)	64.8 (0.7)	F_(1359)_ = 1.39	0.240
Pain	7.7 (0.2)	7.2 (0.2)	F_(1367)_ = 5.81	0.017
KOOS - Pain	31.8 (2.0)	37.1 (1.8)	F_(1364)_ = 6.66	0.010

* All values are least squares means (standard error of the mean) adjusted for sex and race. NOAH cohort models are also adjusted for smoking status.

NOAH = New Orleans Alcohol Use in HIV; SF-36 = 36-Item Short Form Survey; HADS = Hospital Anxiety and Depression Scale; TKA = total knee arthroplasty; PROMIS = Patient-Reported Outcomes Measurement Information System; KOOS = Knee injury and Osteoarthritis Outcome Score.

**Table 4:** Spearman correlations with 36-item short form survey (SF-36) pain intensity and pain interference scores and hospital anxiety and depression scale (HADS) scores for all New Orleans Alcohol Use in HIV (NOAH) participants.

SF-36 Pain Scale Dimension	N	HADS-Anxietyr (p-value)	HADS-Depressionr (p-value)
Pain Intensity	364	−0.264 (<0.0001)	−0.241(<0.0001)
Pain Interference	364	−0.280 (<0.0001)	−0.280 (<0.0001)

SF-36 = 36-Item Short Form Survey; HADS = Hospital Anxiety and Depression Scale

**Table 5:** Spearman correlations with patient-reported outcomes measurement information system (PROMIS-29) pain intensity and pain interference scores and knee injury and osteoarthritis outcome score (KOOS) pain score and PROMIS-29 anxiety and depression scores for all total knee arthroplasty (TKA) participants.

Pain Scale Dimension	N	PROMIS Anxietyr (p-value)	N	PROMIS Depression r (p-value)
PROMIS Pain Interference	357	0.344 (<0.0001)	355	0.380 (<0.0001)
PROMIS Pain Intensity	362	0.338 (<0.0001)	361	0.337 (<0.0001)
KOOS Pain	359	−0.430 (<0.0001)	357	−0.426 (<0.0001)

PROMIS = Patient-Reported Outcomes Measurement Information System; KOOS = Knee Injury and Osteoarthritis Outcome Score.

**Table 6:** Spearman Correlations with 36-Item Short Form Survey (SF-36) pain intensity and pain interference scores and Hospital Anxiety and Depression Scale (HADS) scores by sex and drinking status (AUDIT-C = 0 vs. AUDIT-C ≥ 1) in the New Orleans Alcohol Use in HIV (NOAH) cohort.

SF-36 Pain Scale Dimension& Alcohol Drinking Status	HADS-Anxiety Correlation (r)	HADS-Anxiety p value	z_diff_ p-value^1^	HADS-Depression Correlation (r)	HADS-Depression p value	N	z_diff_ p-value^1^
Intensity (Female Drinkers)	−0.27	0.010	0.897	−0.25	0.016	91	0.865
Intensity (Female Non-Drinkers)	−0.30	0.176		−0.29	0.185	22	
Interference (Female Drinkers)	−0.19	0.073	0.174	−0.27	0.009	91	0.899
Interference (Female Non-Drinkers)	−0.49	0.020		−0.24	0.285	22	
Intensity (Male Drinkers)	−0.25	0.0002	0.761	−0.28	<0.0001	222	0.021
Intensity (Male Non-Drinkers)	−0.19	0.337		0.19	0.316	29	
Interference (Male Drinkers)	−0.28	<0.0001	0.708	−0.32	<0.0001	222	0.090
Interference (Male Non-Drinkers)	−0.35	0.067		0.02	0.936	29	

SF-36 = 36-Item Short Form Survey; HADS = Hospital Anxiety and Depression Scale

1 P-value represents Fisher’s z test, used to compare correlation coefficients between drinkers and non-drinkers.

**Table 7:** Spearman Correlations with Patient-Reported Outcomes Measurement Information System (PROcMIS-29) Pain Intensity and Pain Interference scores and Knee Injury and Osteoarthritis Outcome Score (KOOS) Pain Score and PROMIS-29 Anxiety and Depression scores by sex and drinking status (AUDIT-C = 0 vs. AUDIT-C ≥ 1) in the Total Knee Arthroplasty (TKA) cohort.

Pain Scale Dimension& Alcohol Drinking Status	PROMIS-Anxiety Correlation (r)	PROMIS-Anxiety p value	N	z_diff_ p-value^1^	PROMIS-Depression Correlation (r)	PROMIS-Depression p value	N	z_diff_ p-value^1^
PROMIS Interference (Female Drinkers)	0.378	<0.0001	131	0.826	0.403	<0.0001	129	0.950
PROMIS Interference (Female Non-Drinkers)	0.353	0.0002	110		0.396	<0.0001	108	
PROMIS Intensity (Female Drinkers)	0.376	<0.0001	134		0.401	<0.0001	131	
PROMIS Intensity (Female Non-Drinkers)	0.320	0.0006	113	0.622	0.240	0.0109	112	0.167
KOOS Pain (Female Drinkers)	−0.447	<0.0001	138		−0.487	<0.0001	131	
KOOS Pain (Female Non-Drinkers)	−0.480	<0.0001	111	0.745	−0.480	<0.0001	109	0.945
PROMIS Interference (Male Drinkers)	0.309	0.0032	89		0.411	<0.0001	91	
PROMIS Interference (Male Non-Drinkers)	0.118	0.559	27	0.384	0.037	0.856	27	0.083
PROMIS Intensity (Male Drinkers)	0.328	0.0018	88		0.451	<0.0001	90	
PROMIS Intensity (Male Non-Drinkers)	0.082	0.683	27	0.264	0.058	0.771	28	0.059
KOOS Pain (Male Drinkers)	−0.354	0.0008	87		−0.343	0.001	89	
KOOS Pain (Male Non-Drinkers)	−0.208	0.297	27	0.492	−0.104	0.598	28	0.265

PROMIS = Patient-Reported Outcomes Measurement Information System; KOOS = Knee injury and Osteoarthritis Outcome Score

1 P-value represents Fisher’s z test, used to compare correlation coefficients between drinkers and non-drinkers.

## Data Availability

Data will be made available upon reasonable request.

## Appendix A. Supporting information

Supplementary data associated with this article can be found in the online version at doi:10.1016/j.jpain.2025.105446.
