# Supplementary material for: Intersection of alcohol use, pain symptoms, and negative affect in total knee arthroplasty patients and people with HIV
Source: J Pain. Author manuscript; Available in PMC 2026 May 11. (PMC13159567; doi:10.1016/j.jpain.2025.105446)

**Supplementary Figure 1:** Pain scores stratified by three AUDIT-C categories (non-drinkers: AUDIT-C = 0; low-risk drinkers: AUDIT-C = 1-2 (females) or 1-3 (males); at-risk drinkers: AUDIT-C ≥ 3 (females) or ≥ 4 (males)) among New Orleans Alcohol Use in HIV (NOAH) participants and Total Knee Arthroplasty (TKA) participants. **A.** 36-Item Short Form Survey (SF-36) pain intensity scores did not differ between the three groups. **B.** SF-36 pain interference scores did not differ between the three groups**. C.** Patient-Reported Outcomes Measurement Information System (PROMIS-29) pain intensity scores differed between non-drinkers and low-risk drinkers and between non-drinkers and at-risk drinkers but did not differ between low-risk drinkers and at-risk drinkers. **D.** PROMIS-29 pain interference scores did not differ between the three groups. **E.** Knee Osteoarthritis and Outcomes Score (KOOS) pain scores differed between non-drinkers and low-risk drinkers and between non-drinkers and at-risk drinkers but did not differ between low-risk drinkers and at-risk drinkers.


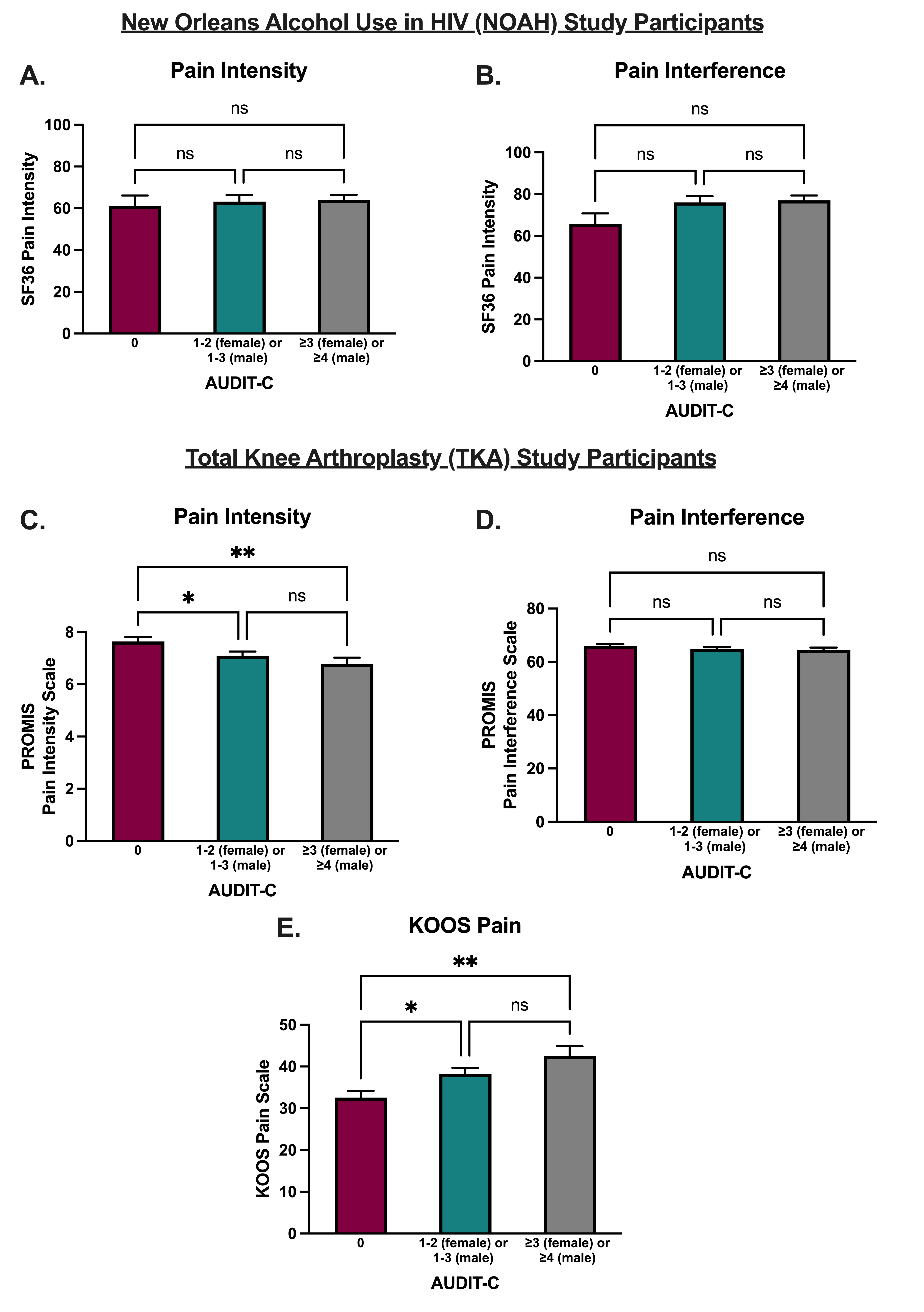

Supplement: Supplementary Figure 1 [file NIHMS2166995-supplement-Supplementary_Figure_1.docx]
