# Supplementary material for: Intersection of alcohol use, pain symptoms, and negative affect in total knee arthroplasty patients and people with HIV
Source: J Pain. Author manuscript; Available in PMC 2026 May 11. (PMC13159567; doi:10.1016/j.jpain.2025.105446)
Supplement: Supplementary Table 1 [file NIHMS2166995-supplement-Supplementary_Table_1.docx]

**Supplementary Table 1:** Exploratory analyses between continuous AUDIT-C and pain variables across NOAH and TKA cohorts.

| **Pain Variable** | **N** | **AUDIT-C**  **Crude**  **Spearman**  **Correlation**  **r (p-value)** | **AUDIT-C**  **Adjusted***  **Spearman**  **Correlation**  **r (p-value)** |
| --- | --- | --- | --- |
| **NOAH cohort** |  |  |  |
| SF-36 Pain Intensity | 364 | 0.033 (0.525) | 0.017 (0.754) |
| SF-36 Pain Interference | 364 | 0.083 (0.115) | 0.066 (0.212) |
| **TKA cohort** |  |  |  |
| PROMIS-29 Pain  PROMIS-29 Pain Interference | 372  364 | -0.165 (0.001)  -0.101 (0.054) | -0.115 (0.035)  -0.054 (0.327) |
| KOOS Pain | 369 | 0.198 (<0.001) | 0.142 (0.009) |

*Adjusted Spearman correlations adjusted for sex and race. NOAH cohort models are also adjusted for smoking status. N=338 for adjusted Spearman correlations in TKA cohort.

NOAH = New Orleans Alcohol Use in HIV; SF-36 = 36-Item Short Form Survey; TKA = total knee arthroplasty; PROMIS = Patient-Reported Outcomes Measurement Information System; KOOS = Knee injury and Osteoarthritis Outcome Score.
